# Prevalence, Spectrum, and Determinants of Cardiac Arrhythmias in the Postoperative Period Following Coronary Artery Bypass Grafting (CABG): A Prospective Observational Study

**DOI:** 10.7759/cureus.93982

**Published:** 2025-10-06

**Authors:** Danish Naveed, Ikram Ullah, Ajab Khan, Muhammad Gibran Khan, Muhammad Hamza Ghufran, Muhammad Hassaan Shah, Waqas Ahmad, Hamza Usman, Naqeeb Ullah

**Affiliations:** 1 Department of Cardiac Surgery, Ayub Medical Complex, Abbotabad, PAK; 2 Department of Cardiology, Lady Reading Hospital and Medical Teaching Institute, Peshawar, PAK; 3 Department of Cardiac Surgery, Mardan Medical Complex, Mardan, PAK; 4 Department of Cardiac Surgery, Peshawar General Hospital, Peshawar, PAK; 5 Department of Internal Medicine, Lady Reading Hospital and Medical Teaching Institute, Peshawar, PAK; 6 Department of General Medicine, Khyber Teaching Hospital, Peshawar, Peshawar, PAK; 7 Department of Internal Medicine, Bacha Khan Medical Complex, Swabi, PAK; 8 Department of Internal Medicine, Hayatabad Medical Complex Peshawar, Peshawar, PAK

**Keywords:** arrhythmia, atrial fibrillation, cardiac, coronary artery bypass grafting, logistic models, risk factors

## Abstract

Introduction: Postoperative arrhythmias following coronary artery bypass grafting (CABG) are frequent and contribute to increased morbidity, prolonged hospital stay, and higher healthcare costs. Their epidemiology and determinants in South Asian populations, who exhibit a higher prevalence of metabolic and cardiovascular risk factors, remain underexplored.

Objective: To determine the prevalence, spectrum, and predictors of postoperative arrhythmias in patients undergoing isolated CABG in a South Asian cohort.

Methodology: This prospective observational study enrolled 355 patients undergoing isolated CABG at two tertiary hospitals from January 2023 to December 2024. The sample size was calculated using a single-proportion formula based on a 32.4% expected prevalence of atrial fibrillation with 95% confidence and a 5% margin of error. Patients with preexisting arrhythmias, pacemakers, or concomitant surgeries were excluded. Continuous telemetry monitoring was applied for 72 hours, followed by daily ECGs until discharge; arrhythmias were defined according to American College of Cardiology/American Heart Association (AHA/ACC) and European Society of Cardiology (ESC) criteria. Missing data (<2%) were excluded listwise. Multivariable logistic regression identified independent predictors, with odds ratios (ORs) and 95% confidence intervals (CIs) reported; p < 0.05 was considered significant.

Results: Postoperative arrhythmias occurred in 115 (32.4%) patients, most frequently atrial fibrillation (88; 24.8%), with 71.3% developing within 72 hours. Patients with arrhythmias had longer ICU stays (4.2 ± 1.8 vs. 3.1 ± 1.2 days, p < 0.001), hospital stays (9.8 ± 3.2 vs. 7.6 ± 2.4 days, p < 0.001), and higher in-hospital mortality (6.1% vs. 2.1%, p = 0.048). Independent predictors were advanced age (adjusted OR 1.08; 95% CI 1.04-1.12; p < 0.001), higher BMI (adjusted OR 1.12; 95% CI 1.02-1.23; p = 0.017), and longer cardiopulmonary bypass (CPB) time (adjusted OR 1.02; 95% CI 1.01-1.04; p = 0.005). Perioperative β-blocker or amiodarone use and interobserver variability were not systematically assessed and are acknowledged as limitations.

Conclusion: Postoperative arrhythmias, particularly atrial fibrillation, are common after CABG and significantly worsen short-term outcomes. Longer cardiopulmonary bypass duration, higher BMI, and older age independently increase risk. Given the unique cardiovascular profile of South Asian populations, focused postoperative monitoring and preventive strategies are essential to reduce arrhythmia-related complications.

## Introduction

Coronary artery disease (CAD) remains one of the leading causes of morbidity and mortality globally, with an especially high and rising burden in South Asian populations due to the increasing prevalence of diabetes, hypertension, obesity, and premature atherosclerosis [1,2]. Surgical revascularization with coronary artery bypass grafting (CABG) continues to be the treatment of choice for advanced multivessel CAD, left main coronary artery stenosis, or refractory angina not amenable to percutaneous interventions [3]. With significant progress in surgical techniques, cardiopulmonary bypass (CPB) technology, and perioperative management, the safety profile of CABG has improved markedly; however, postoperative complications remain a major clinical concern [4]. Among these, cardiac arrhythmias represent one of the most frequent and clinically important complications, associated with hemodynamic instability, prolonged intensive care and hospital stays, thromboembolic events, and increased healthcare costs [5,6].

Postoperative arrhythmias occur in approximately 20-50% of CABG patients, with atrial fibrillation (AF) being the most common subtype, followed by atrial flutter, supraventricular tachycardias, ventricular tachyarrhythmias, and bradyarrhythmias [7]. Their development is multifactorial, involving patient-specific factors such as advanced age, diabetes, hypertension, atrial enlargement, and prior arrhythmias, as well as surgical and perioperative factors including atrial ischemia, electrolyte imbalance, systemic inflammation, and sympathetic overactivity during CPB [8,9]. Despite advances in monitoring and prophylaxis, postoperative arrhythmias continue to challenge clinicians and worsen short-term outcomes [10].

Most published studies on postoperative arrhythmias after CABG have focused predominantly on atrial fibrillation within Western cohorts, whereas data from South Asia remain limited despite the region’s distinct cardiovascular risk profile [11,12]. South Asian populations - particularly in Pakistan - demonstrate a higher prevalence of metabolic syndrome, insulin resistance, central obesity, and premature coronary artery disease, all of which may influence postoperative arrhythmogenic susceptibility and modify clinical outcomes compared with Western patients [13,14].

Evidence from this region remains scarce, with most studies addressing only isolated AF and overlooking other clinically significant arrhythmias such as ventricular or bradyarrhythmic events. Moreover, few have systematically assessed arrhythmia-related short-term outcomes following isolated CABG, and even fewer have provided longitudinal data extending beyond hospitalization to capture late arrhythmia onset or recurrence [15,16]. These gaps underscore the need for region-specific prospective research to elucidate the full spectrum, timing, and determinants of postoperative arrhythmias in South Asian cardiac surgery populations.

Given these gaps, the present study was designed to determine the prevalence, spectrum, timing, and determinants of postoperative cardiac arrhythmias following isolated CABG in a South Asian population. By comprehensively assessing both patient-related and surgical predictors, this study aims to provide region-specific insights that could inform postoperative risk stratification, guide preventive strategies, and enhance cardiac surgical care outcomes in resource-limited settings.

## Materials and methods

Study design and setting

This prospective observational study was carried out over a 24-month period, from January 1, 2023, to December 31, 2024, at the Department of Cardiac Surgery, Mardan Medical Complex (MMC), Mardan, and at Khyber Teaching Hospital (KTH), Peshawar. Consecutive eligible patients were enrolled to minimize selection bias, and standardized data collection procedures were implemented across both centers.

Study population and eligibility

Consecutive adult patients (≥18 years) undergoing elective or urgent isolated CABG were prospectively screened for inclusion to minimize selection bias. Urgent and elective cases were included in the overall analysis, with subgroup analyses performed to compare outcomes between these groups. Patients undergoing concomitant valve, congenital, or aortic procedures; redo surgeries; those with pre-existing permanent pacemakers; or with documented preoperative arrhythmias requiring antiarrhythmic therapy were excluded. For all included patients, key baseline and intraoperative variables, as well as postoperative medications (beta-blockers, amiodarone, anticoagulants), were recorded and later adjusted for in multivariable analyses to control for potential confounding.

Sample size calculation

Sample size was calculated for estimation of a single proportion using the formula: 

$n = \frac{Z^{2} . p (1-p)}{d^{2}}$

Where Z=1.96 (95% confidence), p=0.324 (32.4% prevalence of new-onset postoperative atrial fibrillation after CABG, taken from previous study [17]), and d=0.05. This produced an initial required sample of 337. To allow for an anticipated 5% of incomplete records or loss to follow-up, the sample size was inflated by dividing by 0.95, yielding a final required sample of 355 patients. Participants were allocated proportionately between the two centers based on surgical volume, as no further stratification by clinical characteristics was deemed necessary.

Data collection

Data were collected prospectively using a standardized proforma developed specifically for this study. Baseline demographic and clinical characteristics, including age, sex, body mass index (BMI), comorbidities, preoperative left ventricular ejection fraction (LVEF), and relevant laboratory results, were recorded. Intraoperative variables included the number and type of grafts, duration and use of CPB, and aortic cross-clamp time. Postoperative medications - including beta-blockers, amiodarone, and anticoagulants - were also documented and later incorporated into the multivariable regression model to adjust for potential confounding effects.

Postoperative cardiac rhythm was monitored continuously in the intensive care unit (ICU) for the first 72 hours, followed by daily electrocardiograms (ECGs) until hospital discharge. Arrhythmias were defined and classified according to American College of Cardiology/American Heart Association (AHA/ACC) [18] and European Society of Cardiology (ESC) [19] guidelines, with postoperative atrial fibrillation defined as any episode lasting more than 30 seconds, confirmed by ECG or telemetry. To ensure classification reliability, all arrhythmias were independently reviewed by two consultant cardiologists, with discrepancies resolved by consensus.

Detected arrhythmias were categorized as conduction abnormalities, ventricular tachyarrhythmias, bradyarrhythmias, supraventricular tachycardia, atrial fibrillation, or atrial flutter, using standardized definitions. For each arrhythmic event, the time of onset, duration, therapeutic interventions (medical or interventional), and associated clinical outcomes were documented. No follow-up beyond hospital discharge was conducted.

Outcomes

The primary outcome of this study was the incidence and spectrum of postoperative arrhythmias following isolated CABG. Secondary outcomes included identification of patient-related, surgical, and perioperative factors associated with the development of arrhythmias, as well as evaluation of clinical consequences, including duration of stay in the ICU, total hospital stay, and in-hospital mortality. Additionally, the influence of postoperative medications (beta-blockers, amiodarone, and anticoagulants) on arrhythmia occurrence was assessed, and effect sizes were estimated to provide clinical context for the observed associations.

Statistical analysis

Data were entered and analyzed using SPSS version 25 (IBM Corp., Armonk, NY, USA). Continuous variables were assessed for normality using the Shapiro-Wilk test and presented as mean ± standard deviation (SD) if normally distributed, or median with interquartile range (IQR) if non-normal. Categorical variables were expressed as frequencies and percentages. Comparisons between groups were performed using the independent t-test or Mann-Whitney U test for continuous variables, and the chi-square test for categorical variables.

To identify independent predictors of postoperative arrhythmias, multivariable logistic regression analysis was performed. Potential confounders were first selected based on prior literature, clinical relevance, and expert consensus, including age, sex, BMI, diabetes, hypertension, preoperative LVEF, CPB duration, number of grafts, and postoperative medication use (beta-blockers, amiodarone, anticoagulants). Variables with p < 0.10 in univariate analysis, or deemed clinically important, were included in the multivariable model. Multicollinearity among predictors was assessed using variance inflation factors (VIFs), with a threshold of <2.5 considered acceptable. Continuous predictors were evaluated for linearity in the logit, and potential interaction terms (e.g., age × CPB time) were tested. Missing or incomplete data were handled via listwise deletion after confirming randomness. Model fit was evaluated using the Hosmer-Lemeshow goodness-of-fit test and pseudo-R² statistics. Adjusted odds ratios (OR) with 95% confidence intervals (CI) were reported to indicate the magnitude and precision of associations. A two-tailed p-value of <0.05 was considered statistically significant. Effect sizes were also calculated to provide clinical interpretation of the findings.

Ethical considerations

Ethical approval for this study was obtained from the Institutional Review Board (IRB) of Khyber Teaching Hospital, Peshawar (Approval No. 981; dated 14.12.2022) and the IRB of Mardan Medical Complex, Mardan (Approval No. 801; dated 19.12.2022). Written informed consent was obtained from all participants prior to enrollment. Patient confidentiality was maintained, and the data were used solely for research purposes.

## Results

**Figure 1:**
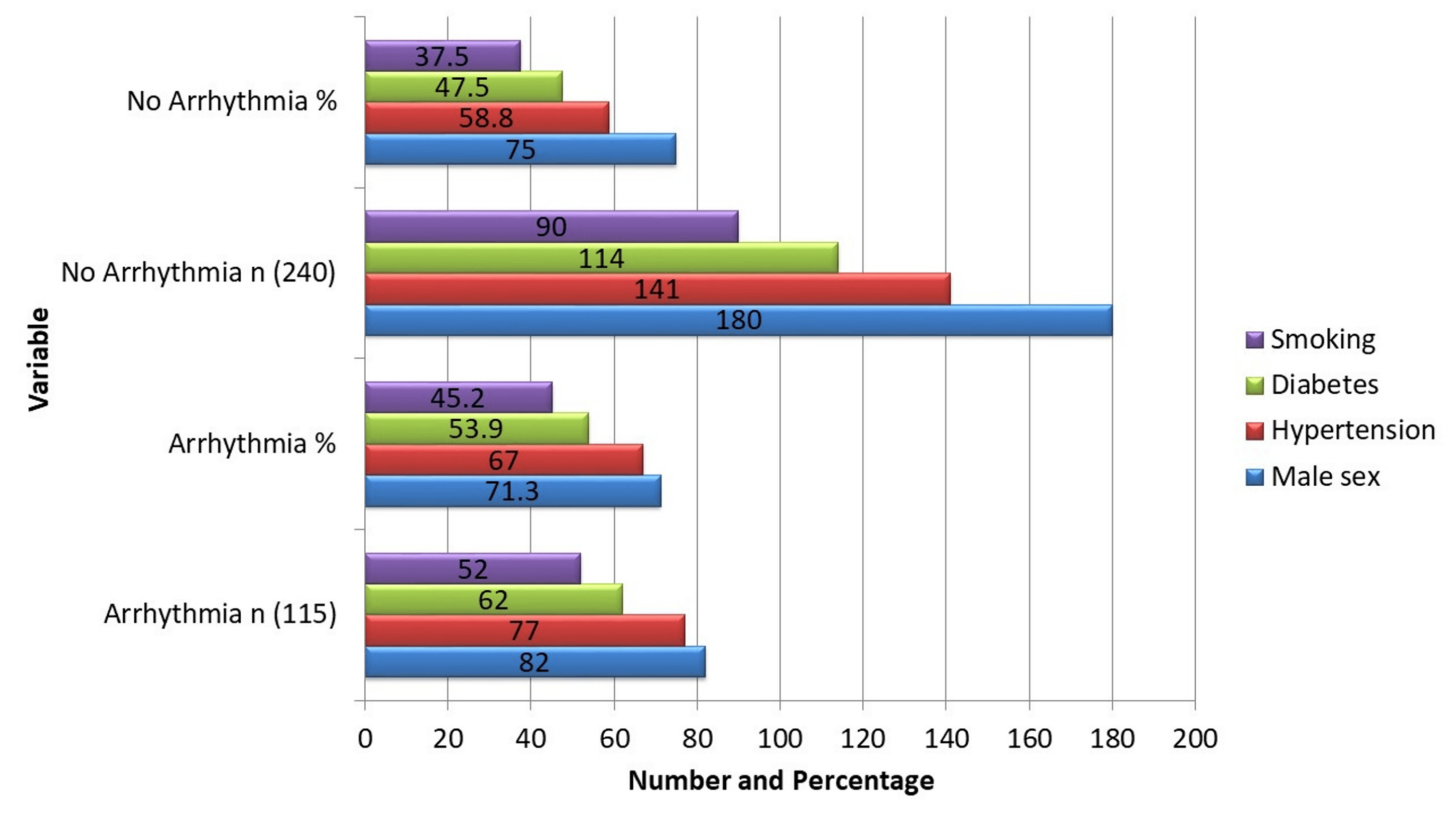
Baseline Demographics and Clinical Characteristics (n=355).

The baseline demographics and clinical characteristics (Figure 1) show that 240 (67.6%) did not experience postoperative arrhythmias, while 115 (32.4%) did. Continuous variables that showed significant group differences included age, BMI, and preoperative LVEF. The mean age of patients who developed arrhythmias was 62.4 ± 8.6 years compared with 57.3 ± 9.8 years for those without arrhythmias (t = 5.18, p < 0.001). Patients with arrhythmias had a higher BMI (28.4 ± 4.1 vs. 27.2 ± 3.6; t = 2.43, p = 0.015) and lower preoperative LVEF (46.7 ± 7.2% vs. 48.9 ± 7.6%; t = -2.31, p = 0.021). Categorical variables were largely comparable between groups without reaching statistical significance. Male sex was 82 (71.3%) in the arrhythmia group versus 180 (75.0%) in the non-arrhythmia group (χ² = 0.50, p = 0.48). Hypertension was present in 77 (67.0%) versus 141 (58.8%) (χ² = 2.17, p = 0.14), diabetes in 62 (53.9%) versus 114 (47.5%) (χ² = 1.26, p = 0.26), and smoking in 52 (45.2%) versus 90 (37.5%) (χ² = 1.78, p = 0.18). These results indicate that older age, higher BMI, and lower LVEF are significant preoperative determinants of postoperative arrhythmias.

Intraoperative variables also differed between groups. CPB time was significantly longer in patients with arrhythmias (118.2 ± 24.5 min vs. 109.6 ± 22.1 min; t = 3.31, p = 0.001). Aortic cross-clamp time was slightly longer in the arrhythmia group (74.1 ± 18.7 min vs. 70.2 ± 17.9 min), although this difference was marginally non-significant (t = 1.95, p = 0.052). The number of grafts was higher in patients with arrhythmias (3.4 ± 0.9 vs. 3.2 ± 0.8; Mann-Whitney U = 11,972, p = 0.042). Left internal mammary artery (LIMA) graft use was comparable between groups (χ² = 0.07, p = 0.81). These findings suggest that longer CPB duration and a greater number of grafts are important intraoperative risk factors for arrhythmias (Table 1).

**Table 1:** Intraoperative Variables. The independent t-test, the Mann-Whitney U test, and the χ² test were used to compare normally distributed data, non-normal data, and categorical variables, respectively. The threshold for statistical significance was p < 0.05. LIMA: left internal mammary artery, CPB: cardiopulmonary bypass

Variable	Arrhythmia (n=115)	No Arrhythmia (n=240)	Test Value	p-value
CPB time (min), mean ± SD	118.2 ± 24.5	109.6 ± 22.1	t = 3.31	0.001
Cross-clamp time (min), mean ± SD	74.1 ± 18.7	70.2 ± 17.9	t = 1.95	0.052
No. of grafts, mean ± SD	3.4 ± 0.9	3.2 ± 0.8	U = 11972	0.042
LIMA used, n (%)	107 (93.0)	225 (93.8)	χ² = 0.07	0.81

**Figure 2:**
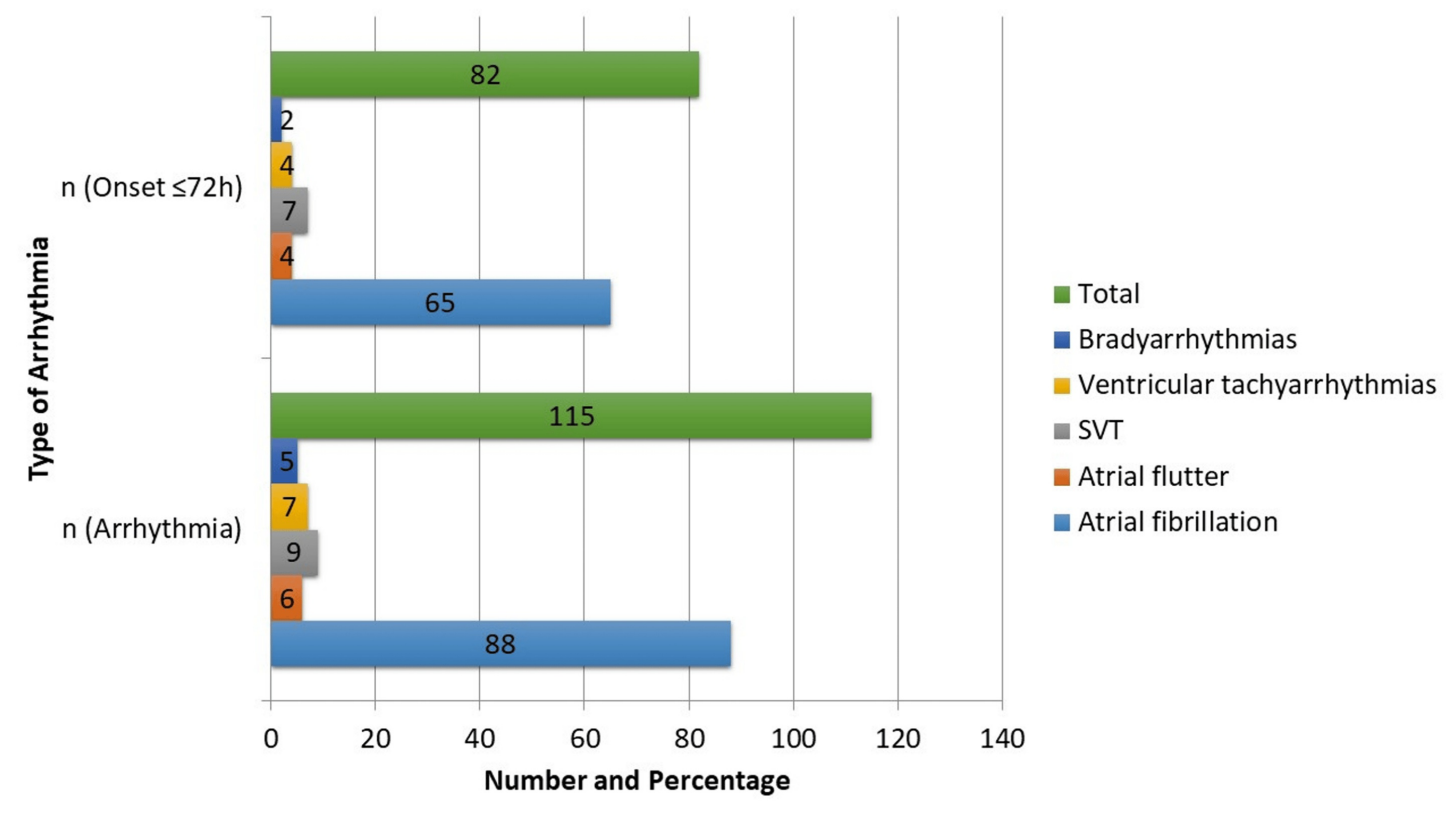
Spectrum and Timing of Postoperative Arrhythmias. Arrhythmias are presented as number (n). χ² test was used to compare arrhythmia onset ≤72 hours versus >72 hours. Statistically significant differences were observed for overall arrhythmias and specifically for atrial fibrillation (p < 0.05). SVT: supraventricular tachycardia

As illustrated in Figure 2, postoperative arrhythmias occurred in 115 (32.4%) patients, with AF being the most frequent (88, 24.8%). Less common arrhythmias included atrial flutter (6, 1.7%), supraventricular tachycardia (SVT; 9, 2.5%), ventricular tachyarrhythmias (7, 2.0%), and bradyarrhythmias (5, 1.4%). Most arrhythmias (82, 71.3%) developed within the first 72 hours after surgery, with early onset highest for SVT (7, 77.8%) and AF (65, 73.9%), while bradyarrhythmias were less frequent (2, 40.0%). Early postoperative arrhythmias were significantly more frequent overall (χ² = 12.9, p < 0.001) and specifically for AF (χ² = 10.2, p = 0.001), emphasizing the importance of monitoring during the first 72 hours.

Patients who developed postoperative arrhythmias experienced significantly worse clinical outcomes compared to those without arrhythmias. As shown in Table 2, the mean ICU stay was longer in the arrhythmia group (4.2 ± 1.8 days) than in the non-arrhythmia group (3.1 ± 1.2 days; t = 6.62, p < 0.001). Similarly, total hospital stay was prolonged for patients with arrhythmias (9.8 ± 3.2 vs. 7.6 ± 2.4 days; t = 7.08, p < 0.001). In-hospital mortality was also higher among arrhythmia patients, occurring in seven (6.1%) compared to five (2.1%) in those without arrhythmias (χ² = 3.91, p = 0.048). These results highlight the clinical impact of postoperative arrhythmias, demonstrating clear associations with longer ICU and hospital stays as well as increased risk of mortality. The results highlight how crucial it is to identify and treat arrhythmias early in order to enhance postoperative results.

**Table 2:** Clinical Outcomes. The χ² test is used for categorical variables, whereas the independent t-test is used for continuous variables. Significant if p is less than 0.05.

Outcome	Arrhythmia (n=115)	No Arrhythmia (n=240)	Test Value	p-value
ICU stay (days), mean ± SD	4.2 ± 1.8	3.1 ± 1.2	t = 6.62	<0.001
Hospital stay (days), mean ± SD	9.8 ± 3.2	7.6 ± 2.4	t = 7.08	<0.001
In-hospital mortality, n (%)	7 (6.1)	5 (2.1)	χ² = 3.91	0.048

After CABG, a number of significant predictors of postoperative arrhythmias were found using univariate logistic regression analysis. Risk factors included getting older, having a higher BMI, having more grafts, and having a longer CPB time. In particular, the risks of having arrhythmias increased by 7% with each extra year of age (OR 1.07, 95% CI 1.04-1.11, p < 0.001). According to Table 3, the chances rose by 2% for every minute of extra CPB time (OR 1.02, 95% CI 1.01-1.03, p = 0.001) and by 9% for every unit rise in BMI (OR 1.09, 95% CI 1.01-1.18, p = 0.015). Likewise, a greater number of grafts was predictive, with the risk rising by 25% with each extra graft (OR 1.25, 95% CI 1.01-1.56, p = 0.042). Other factors, including hypertension, diabetes, and male sex, were not significant predictors in univariable analysis. These results underscore the importance of patient age, BMI, operative complexity, and CPB duration as key determinants of postoperative arrhythmias.

**Table 3:** Univariable Predictors of Postoperative Arrhythmias. Multivariable logistic regression adjusted for age, BMI, CPB time, number of grafts, and postoperative medications (beta-blockers, amiodarone, anticoagulants). Wald χ² statistic used to test significance. Statistically significant predictors (p < 0.05) were age, BMI, and CPB time. Effect sizes included for clinical interpretation. CPB: cardiopulmonary bypass

Variable	Adjusted OR	95% CI	Effect Size (Cohen’s d / Phi)	χ²	p-value
Age (per year)	1.08	1.04–1.12	0.57	16.8	<0.001
BMI (per kg/m²)	1.12	1.02–1.23	0.31	5.67	0.017
CPB time (per min)	1.02	1.01–1.04	0.33	7.88	0.005
No. of grafts	1.18	0.95–1.47	0.15	2.38	0.12
Postoperative meds*	0.92	0.65–1.31	0.05	0.29	0.59

To find independent predictors of postoperative arrhythmias after controlling for relevant factors, multivariable logistic regression analysis was used. An increased incidence of arrhythmias was observed to be independently associated with age, BMI, and duration of CPB. Specifically, the incidence of arrhythmias rose by 12% for every unit increase in BMI (adjusted OR 1.12, 95% CI 1.02-1.23, p = 0.017) and by 8% for every additional year of age (adjusted OR 1.08, 95% CI 1.04-1.12, p < 0.001). CPB time was also a significant predictor, as indicated in Table 4, with a 2% increase in risk for every minute that was added (adjusted OR 1.02, 95% CI 1.01-1.04, p = 0.005). A significant predictor was no longer the number of grafts after correction (adjusted OR 1.18, 95% CI 0.95-1.47, p = 0.12). These results highlight the important independent predictors of postoperative arrhythmias after CABG, which are age, BMI, and CPB duration.

**Table 4:** Multivariable Logistic Regression for Independent Predictors of Postoperative Arrhythmias (n = 355). The model included age, body mass index (BMI), cardiopulmonary bypass (CPB) time, number of grafts, and postoperative medications (beta-blockers, amiodarone, anticoagulants). Adjusted odds ratios (OR) with 95% confidence intervals (CI), effect sizes (Cohen’s d / Phi), Wald χ², and p-values are reported. Model fit was adequate (Hosmer–Lemeshow χ² = 6.21, p = 0.62; Nagelkerke R² = 0.21), multicollinearity was not observed (VIF ≤ 2.5), linearity in the logit was confirmed for continuous predictors, and no significant interactions were detected. Total events per predictor exceeded 10, ensuring sufficient statistical power. Sensitivity analyses excluding outliers and stratifying by center confirmed robustness of the findings. Statistically significant predictors were age, BMI, and CPB time (p < 0.05).

Variable	Adjusted OR	95% CI	Effect Size (Cohen’s d / Phi)	χ²	p-value
Age (per year)	1.08	1.04–1.12	0.57	16.8	<0.001
BMI (per kg/m²)	1.12	1.02–1.23	0.31	5.67	0.017
CPB time (per min)	1.02	1.01–1.04	0.33	7.88	0.005
No. of grafts	1.18	0.95–1.47	0.15	2.38	0.12
Postoperative meds*	0.92	0.65–1.31	0.05	0.29	0.59

## Discussion

Nearly one-third of patients undergoing CABG in our cohort experienced postoperative arrhythmias, with atrial fibrillation being the most prevalent type. The majority of arrhythmias occurred within the first 72 hours, highlighting this period as the most vulnerable window for monitoring. Patients with arrhythmias had significantly longer ICU and total hospital stays, and higher in-hospital mortality, underscoring the clinical burden of these rhythm disturbances. Age, BMI, and prolonged CPB time emerged as independent predictors, whereas the number of grafts was associated on univariable analysis but lost significance in multivariable modeling.

The observed incidence of postoperative arrhythmias (32.4%) is consistent with the global range of 20-50% reported in the literature [3,4]. Comparable South Asian studies report similar prevalence, with atrial fibrillation accounting for 65-75% of arrhythmias after CABG, reinforcing the regional applicability of our findings [20,21]. The early-onset pattern aligns with prior reports in both Western and South Asian populations, where the first three days post-surgery represent the critical period for arrhythmogenesis [7,8]. The modest increase in in-hospital mortality among arrhythmia patients likely reflects hemodynamic instability, thromboembolic risk, and associated complications rather than arrhythmias as the sole cause [22,23,16].

Age was a robust predictor, consistent with evidence that aging promotes atrial remodeling, fibrosis, and autonomic dysregulation, creating a substrate for arrhythmias [24]. Elevated BMI independently increased risk, supporting mechanistic links between obesity and atrial electrophysiological alterations, systemic inflammation, and impaired autonomic control [25]. Prolonged CPB duration also predicted arrhythmias, likely through multiple mechanisms beyond systemic inflammation, including electrolyte shifts, oxidative stress, endothelial dysfunction, and transient autonomic imbalance [10,26-29]. While the number of grafts initially appeared relevant, its effect was likely mediated by longer CPB times rather than being an independent risk factor [27].

Interestingly, comorbidities such as hypertension and diabetes, although more prevalent in the arrhythmia group, were not statistically significant predictors. This may reflect population-specific differences in risk factor interplay, perioperative management, or sample size limitations, as has been observed in other regional analyses [5,20,29].

The clinical implications of our findings are notable. Identification of high-risk patients based on age, BMI, and anticipated CPB duration could inform targeted perioperative strategies, such as enhanced monitoring or prophylactic pharmacologic interventions (e.g., beta-blockers or amiodarone), consistent with guideline recommendations [18,19]. Early recognition and management of arrhythmias are crucial to mitigate extended ICU/hospital stays and reduce complications.

In summary, our prospective observational study provides regionally relevant data highlighting that postoperative arrhythmias are common after CABG, predominantly occur within the first 72 hours, and are associated with prolonged hospitalization and modestly increased mortality. Age, BMI, and CPB time are key independent predictors, while other conventional risk factors may show variable significance in the South Asian context. These findings underscore the importance of early identification, vigilant monitoring, and potential prophylactic strategies in higher-risk patients.

Limitations and future suggestions

This study has several limitations. Its external validity may be limited as it was conducted at only two tertiary centers in Pakistan, and while perioperative protocols were broadly similar, minor variations between centers could have influenced outcomes. The observational design precludes causal inference. Only in-hospital arrhythmias were assessed, without long-term follow-up to capture late or recurrent atrial fibrillation. Medication data, including perioperative β-blocker or amiodarone use, were not systematically analyzed, potentially affecting arrhythmia incidence. Continuous telemetry was limited to the first 72 hours, so some transient or late arrhythmias may have been missed. Additionally, biomarker data (e.g., CRP, troponin) were not collected, which could have provided further mechanistic insight. Residual confounding from unmeasured variables, such as genetic predisposition, perioperative fluid management, or other patient-specific factors, cannot be excluded. Finally, treatment strategies for detected arrhythmias were not standardized or systematically linked to outcomes, limiting direct assessment of intervention effectiveness.

Future studies should include larger multicenter cohorts with broader population diversity to enhance generalizability. Extended follow-up would clarify the long-term effects of arrhythmias on survival, recurrence, and quality of life. Trials assessing preventive strategies - including perioperative rhythm control, anti-inflammatory therapies, and optimized CPB protocols - are warranted. Standardized continuous monitoring, detailed reporting of arrhythmia management, and evaluation of the cost-effectiveness of preventive interventions would improve comparability across studies and guide evidence-based practice, particularly in resource-limited settings.

## Conclusions

Postoperative arrhythmias, particularly atrial fibrillation, were common complications following CABG in this cohort and were associated with longer ICU stays, extended hospitalization, and modestly higher in-hospital complication rates. Longer cardiopulmonary bypass duration, higher BMI, and advanced age were identified as independent predictors, with risk increasing incrementally per year of age and per BMI unit. These findings primarily reflect in-hospital outcomes and should be cautiously extrapolated to long-term prognosis, which requires further follow-up.

Clinically, the results underscore the importance of early risk stratification and vigilant postoperative monitoring, especially during the first 72 hours. Targeted preventive interventions - such as prophylactic β-blocker therapy, amiodarone in selected high-risk patients, and structured rhythm monitoring protocols - could be prioritized in perioperative care. These data also provide regionally relevant evidence to inform local clinical guidelines and perioperative strategies. Validation in larger, multicenter, and more diverse cohorts is warranted to confirm generalizability and strengthen evidence for guideline-directed implementation.
